# Cervical Cancer Screening Barriers and Risk Factor Knowledge Among Uninsured Women

**DOI:** 10.1007/s10900-017-0316-9

**Published:** 2017-02-02

**Authors:** Marvellous Akinlotan, Jane N. Bolin, Janet Helduser, Chinedum Ojinnaka, Anna Lichorad, David McClellan

**Affiliations:** 1 Department of Health Policy & Management, Texas A&M School of Public Health, TAMU 1266, College Station, TX 77843-1266 USA; 2 Department of Health Sciences, University of Missouri, Columbia, MO 65211 USA; 3 Department of Clinical Translational Medicine, College of Medicine, Texas A&M Health Science Center, 2900 E. 29th Street, Bryan, TX 77802 USA

**Keywords:** Cervical cancer, Screening, Knowledge, Barriers, Uninsured

## Abstract

A steady decline in cervical cancer incidence and mortality in the United States has been attributed to increased uptake of cervical cancer screening tests such as Papanicolau (Pap) tests. However, disparities in Pap test compliance exist, and may be due in part to perceived barriers or lack of knowledge about risk factors for cervical cancer. This study aimed to assess correlates of cervical cancer risk factor knowledge and examine socio-demographic predictors of self-reported barriers to screening among a group of low-income uninsured women. Survey and procedure data from 433 women, who received grant-funded cervical cancer screenings over a span of 33 months, were examined for this project. Data included demographics, knowledge of risk factors, and agreement on potential barriers to screening. Descriptive analysis showed significant correlation between educational attainment and knowledge of risk factors (r = 0.1381, P < 0.01). Multivariate analyses revealed that compared to Whites, Hispanics had increased odds of identifying fear of finding cancer (OR 1.56, 95% CI 1.00–2.43), language barriers (OR 4.72, 95% CI 2.62–8.50), and male physicians (OR 2.16, 95% CI 1.32–3.55) as barriers. Hispanics (OR 1.99, 95% CI 1.16–3.44) and Blacks (OR 2.06, 95% CI 1.15–3.68) had a two-fold increase in odds of agreeing that lack of knowledge was a barrier. Identified barriers varied with age, marital status and previous screening. Programs aimed at conducting free or subsidized screenings for medically underserved women should include culturally relevant education and patient care in order to reduce barriers and improve screening compliance for safety-net populations.

## Introduction

Over the past four decades, there has been a steady decline in cervical cancer incidence and mortality in the United States [1–3]. This decline has been largely attributed to increased uptake of cervical cancer screening tests such as Papanicolau (Pap) tests [2]. The U. S. Preventive Task Force recommends that women between the ages of 21 and 65 obtain a Pap test every 3 years [4]. Despite this evidence, sociodemographic disparities in screening still exist [5, 6]. Cervical cancer screening rates vary by socio-demographic factors such as race and ethnicity [6, 7], educational attainment [6–8], income [5, 6], health insurance [9], and immigration status [5, 7, 10].

Previous studies focusing on socio-demographic disparities in cervical cancer screening found that Hispanics are less likely to have had previous Pap tests, compared to non-Hispanic Blacks or non-Hispanic Whites [6, 11]. Other studies found that older minority women were less likely to be screened compared to their white counterparts, leading to late stage diagnosis of cervical cancer [8, 12, 13]. Also, women who have low income, and low educational attainment are less likely to be screened, or follow up with results of abnormal cytology [5]. In terms of access, several studies have shown that lack of health insurance and not having a regular source of primary health care are major barriers to screening [6, 7, 14, 15].

There are also geographical variations in access and uptake of cervical cancer screening, with rural women less likely to be screened for cervical cancer compared to urban and suburban women [2, 16]. These observed disparities may be due to fewer primary care physicians in rural areas and higher population of uninsured residents. Lower screening rates have been reported for the southern part of the United States, which reportedly has the highest proportion of women who have not been screened in the past five years [14]. In Texas, improving women’s cancer prevention services (screening and education) in rural areas and among health-disparate populations has been the focus of significant and prolonged concern. Texas ranks 48th for Pap test screening compliance, with screening rates of 80.3% among non-Hispanic whites and 75.7% among Hispanics [17]. Based on data from the Behavioral Risk Factor Surveillance System (BRFSS), across the United States the average screening rate for women ages 21 to 65 years was 82.6% in 2014, and 77.7% in Texas [17].

Barriers to cervical cancer screening contribute to disparities in cervical cancer screening rates. These barriers have been broadly divided into personal and structural impediments [18, 19]. Personal barriers explored in literature include fear of finding cancer [8, 18], embarrassment [18], lack of knowledge of risk factors [20, 22], screening by a male physician [18], recent immigration status [5, 10], and presence of chronic diseases [23]. Other studies have examined structural barriers such as cost [6, 18], taking time off work [18], lack of transportation [18], poor English proficiency [10, 18], fewer routine physician visits [10, 13], lack of child care [18], and lack of physician recommendation [12].

Even when some of these structural barriers are removed through the provision of free screening programs, some low-income women fail to take advantage of these opportunities [24]. Therefore, better understanding of the barriers and misconceptions about the risk factors for cervical cancer among this population is needed. The purpose of this study was to assess the correlates of cervical cancer risk factor knowledge and predictors of perceived structural and personal barriers to screening among a group of uninsured or low-income underinsured women.

## Materials and Methods

### Population and Sample

Through a grant from the Cancer Prevention and Research Institute of Texas (CPRIT), women who met financial eligibility requirements received free or subsidized cervical cancer screening services through the Texas Cancer Screening, Training, Education and Prevention Program, or Texas C-STEP. Uninsured women with household incomes below 250% of federal poverty level, who were over 21 years of age with no prior history of hysterectomy for benign disease were eligible to receive the free cervical cancer screening services at a university-affiliated family medicine center. Community health workers assisted in determining financial eligibility, scheduling appointments, instructing patients, and completing a new patient application packet including an intake survey. Patients were consented according to the clinic’s operating procedures. Analysis of the de-identified intake survey and procedural data were approved by the university’s institutional review board (IRB) under TAMU IRB# 2013-0855D.

The intake survey assessed the following domains: demographics, personal and family history of cervical cancer; history of previous Pap tests and results, knowledge of risk factors of cervical cancer, and potential barriers associated with receiving a Pap test. Spanish-speaking participants who opted to communicate in Spanish were assigned to bilingual community health workers who administered the survey in Spanish. Patient data were de-identified before analysis by non-clinical university personnel.

Demographic variables included age, ethnicity, race, marital status, education, income, and employment status. However, income was excluded in the analysis due to poor accuracy of answers provided on the survey and low variation in responses. Patient knowledge about risk factors for cervical cancer was assessed using a 10-item true or false questionnaire, with scores for total number of correct responses ranging from zero to ten (higher scores represent better knowledge about cervical cancer risk factors). The internal consistency of the knowledge scale was 0.75. A 10-item scale developed by Champion et al. [25] was used to measure the participants’ perception of potential barriers to having a Pap test. Responses to the questionnaire were graded on a 5-point Likert scale, with responses ranging from 1 (strongly disagree) to 5 (strongly agree). Items captured the main categories of barriers identified in prior scientific studies [18, 19]: that is, personal barriers including feelings of embarrassment, fear of finding cancer, anxiety about the procedure, lack of knowledge, anticipation of pain, other health problems, language barriers; and structural barriers such as cost, transportation, lack of time, male physician. The internal consistency (Cronbach alpha) of the barriers scale was 0.84.

### Statistical Analysis

Descriptive statistics were calculated for socio-demographic characteristics, knowledge of cervical cancer, and barriers to cervical cancer screening. For analysis of the barriers items, we collapsed participants’ responses to the 5-point Likert scale into three categories: 1 (disagree), 2 (neither agree or disagree) and 3 (agree). We assessed the correlates of accurate risk factor knowledge and used Chi square tests to measure bivariate associations between barriers to cervical cancer screening by race. Separate multivariate logistic regression models were used to estimate the odds of identifying each barrier to cervical cancer screening. Independent variables chosen were race/ethnicity, marital status, education, age, and previous cervical cancer screening. These covariates were included in the regression analyses based on *a priori* hypotheses [6, 8, 11–14]. Due to limited sample size of other racial groups, only respondents who self-reported as White, Black, or Hispanic where included in the bivariate and multivariate analysis. The alpha level for statistical significance was set at 0.05. STATA 14.1 was used for all statistical analysis (StataCorp, College Station, TX) [26].

## Results

### Demographic Description of Sample

Five hundred and twenty-four women from 17 counties in Texas received grant-funded cervical cancer screening and diagnostic services over a span of 33 months. Surveys were administered during the first clinic visit, before any screening services were received, and the survey participation rate was 81% (n = 433). Sociodemographic characteristics of the study sample are presented in Table 1. More than of the respondents were between 30 and 49 years of age (51.8%). About forty-one percent reported their ethnicity as Hispanic, 25.9% reported race as Black, and 31% as White. More than three-quarters had a high school education or less, and about 62% were single. Ninety-five percent of the women were uninsured, and almost half (46%) had no regular source of primary health care. Sixty-nine women (15.9%) reported individual or family histories of cervical cancer. Forty-six percent of the respondents had not been screened within the past 2 years.

**Table 1:** Sociodemographic characteristics of cervical cancer screening recipients (n = 433)

Variables	No.	%
Age (years)		
18–29	87	20.1
30–49	220	51.8
50 and older	123	28.4
Missing	3	0.7
Race/ethnicity		
Non-Hispanic White	134	31.0
Black	112	25.9
Hispanic	176	40.7
Other	1	0.2
Missing	10	2.3
Education		
High school diploma or less	358	82.7
Some college	58	13.4
College graduate or more	9	2.1
Missing	8	1.9
Marital status		
Married	137	31.6
Single	268	61.9
Living with Partner	9	2.1
Widowed	1	0.2
Missing	18	4.2
Insurance Status		
Uninsured	415	95.8
Medicaid	10	2.3
Commercial Insurance	6	1.4
Missing	2	0.5
Family/individual history of cervical cancer		
No	296	68.4
Yes	69	15.9
Not sure	48	11.1
Missing	20	4.6
Regular source of care		
No	199	46.0
Yes	224	51.7
Missing	10	2.3
Primary language spoken		
English	328	75.6
Spanish	102	23.6
Previous Pap test*		
Yes	229	52.9
No	102	46.7
Missing	2	0.5

*Defined as having had a Pap test within the past 2 years

### Knowledge About Cervical Cancer

Table 2 shows the proportion of correct responses to the ten knowledge measures. Overall, 3.2% of respondents were not aware of any of the risk factors; that is, 3.2% of the respondents answered all true–false questions incorrectly. Among participants who accurately identified one or more risk factors, more than 70% were aware that a woman is at higher risk of cervical cancer if she has unprotected sex or has a sexually transmitted disease or virus; but only 60.5% were aware that multiple sexual partners posed a risk. About two-thirds recognized a compromised immune system as a risk factor (64.4%).

**Table 2:** Correct responses to questions about risk of cervical cancer

Correct responses	
A woman is more likely to have cancer if …	n = 433 (%)
… she does not go for regular (Pap) smears/tests	77.4
… it runs in her family	75.3
… she has unprotected sex	73.0
… she has a sexually transmitted disease or virus	71.8
… she has a weakened immune system	64.4
… she has had many sexual partners	60.5
… she started having sex at a young age	51.5
… she smokes cigarettes	49.0
… she used birth control pill for a long time	36.7
… she has many children	23.3

More than three-quarters of the participants recognized the need to go for regular Pap tests for early detection of cervical cancer (77.4%), but were less aware of the higher risk of cervical cancer due to having sex at a young age (51.5%), or smoking cigarettes (49%). Family history was more frequently recognized as a risk factor (75%) compared to long-term use of birth control (36.7%) and multiple births (23.3%).

In general, only eight percent of the study sample identified all ten risk factors for cervical cancer. The average score was six out of the ten risk factors (60% correct responses) on the survey. There was little variation by race and ethnicity in the number of risk factors identified. The average risk factor knowledge score was 6.0 for Whites, 6.1 for Blacks, and 5.5 out of 10 for Hispanics; however, this difference was not statistically significant (P = 0.148). There were no significant correlations between age and risk factor knowledge (r = 0.016, P = 0.736), or marital status and risk factor knowledge (r = 0.037, P = 0.459), or previous Pap screening and risk factor knowledge (r = 0.069, P = 0.151). However, there was a significant positive correlation between educational attainment and knowledge of risk factors (r = 0.1381, P < 0.01).

### Identified Barriers to Receiving a Pap Test

**Fig. 1:**
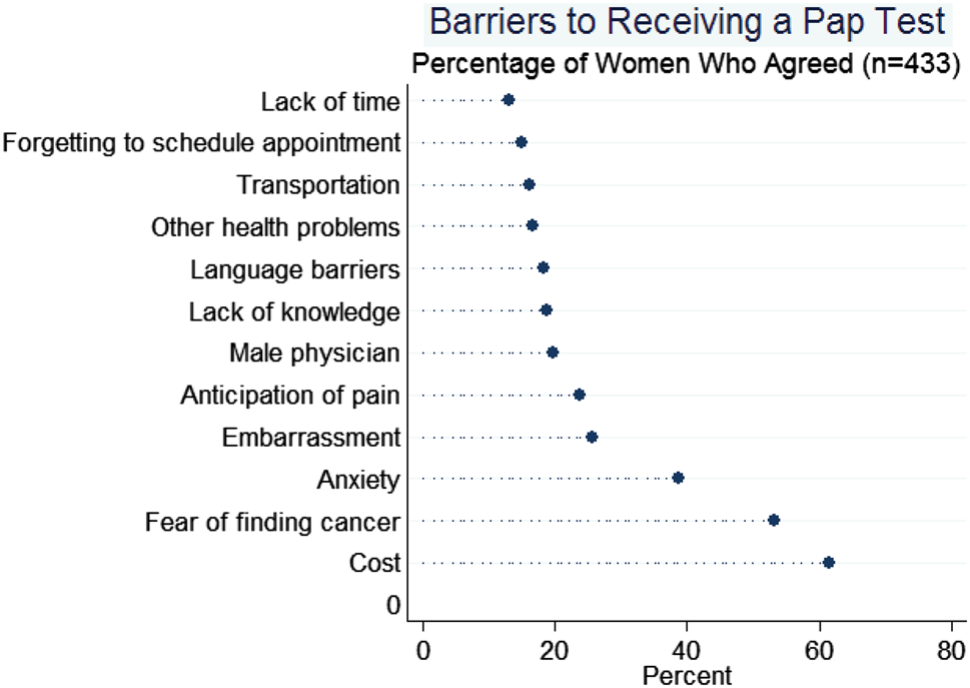
Respondents’ perception of barriers to Pap testing

Figure 1 shows the percentage of women who agreed or strongly agreed with each of the items presented as potential barriers to receiving a Pap test. Not surprisingly, a majority of respondents identified cost as a barrier to receiving a Pap test (61.6%). More than half of the respondents (53.1%) agreed that finding cancer was a barrier to Pap screening. Anxiety about the procedure was the third most commonly agreed-upon barrier (38.7%). Feelings of embarrassment (25.6%), anticipation of pain (23.6%), and the presence of a male physician (19.7%) were identified as barriers by one-quarter or less of the women. Fewer than 20% identified lack of knowledge (18.8%), language barriers (18.3%), and other health problems (16.5%) as potential hindrances to cervical cancer screening. Forgetting to schedule an appointment (14.9%), and lack of time (13%) were identified as barriers by relatively few of the participants. Overall, 15% of the respondents agreed that all the items were barriers, and only 7% disagreed that any items were barriers .

Chi square tests were used to examine the bivariate associations between identified barriers to cervical cancer screening and race/ethnicity. Table 3 shows the results of the Chi square tests. With regards to race and ethnicity, Blacks were more likely to report other health problems (*P* value = 0.04), and lack of knowledge about cervical cancer (*P* value = 0.004), as barriers to being screened. About 37% of Hispanics identified language as a barrier, compared to 7.4% of Whites and 3.5% of Blacks (*P* < 0.001). Similarly, Hispanics (26%) were more likely to identify male physicians as barriers compared to Whites (19.4%) and Blacks (11.4%) (*P* < 0.001).

**Table 3:** Barriers to receiving pap tests by race and ethnicity

Barriers	White	Black	Hispanic	*P* value
% agreeing with barrier	N = 132 (%)	N = 114 (%)	N = 177 (%)	
Feelings of embarrassment	25.8	22.8	29.4	0.548
Fear of finding cancer	43.9	58.8	57.1	0.149
Anxiety about procedure	34.9	43.0	40.7	0.173
Anticipation of pain	22.0	29.0	22.0	0.608
Lack of knowledge	9.1	27.2	19.8	0.002
Forgetting to schedule an appointment	19.7	17.5	10.2	0.071
Other health problems	19.7	21.1	11.9	0.046
Male physician	19.7	11.4	26.0	0.000
Cost	69.0	59.7	57.6	0.140
Transportation	14.4	19.3	14.7	0.726
Lack of time	15.9	14.9	9.6	0.667
Language barriers	7.6	3.5	37.3	0.000

Using multivariate analyses, we explored the socio-demographic determinants of variation in barriers to cervical cancer screening. Only observations with complete data were included in the multivariate analyses (n = 396). Results from models with significant findings are presented in Table 4. Barriers such as fear of finding cancer, lack of knowledge, language barriers, and male physicians demonstrated significant racial variation. Compared to whites, Hispanics had increased odds of identifying fear of finding cancer (OR 1.56, 95% CI 1.00–2.43), language barriers (OR 4.72, 95% CI 2.62–8.50), and male physicians (OR 2.16, 95% CI 1.32–3.55) as barriers. Hispanics (OR 1.99, 95% CI 1.16–3.44) and Blacks (OR 2.06, 95% CI 1.15–3.68) had a two-fold increase in the odds of agreeing that lack of knowledge was a barrier.

**Table 4:** Multivariate analyses of self-reported barriers

Independent variables	Feeling embarrassed		Fear of finding cancer		Lack of knowledge		Forgetting to schedule appointment	
	Adjusted OR (95% CI)	*P* value	Adjusted OR (95% CI)	*P* value	Adjusted OR (95% CI)	*P* value	Adjusted OR (95% CI)	*P* value
Race/ethnicity								
White	1.0 (ref)		1.0 (ref)		1.0 (ref)		1.0 (ref)	
Black	0.91 (0.52-1.57)	0.729	1.43 (0.88–2.32)	0.145	2.06 (1.15–3.68)	0.015	0.66 (0.37–1.23)	0.194
Hispanic	1.45 (0.88–2.38	0.147	1.56 (1.00-2.43)	0.048	1.99 (1.16–3.44)	0.013	0.66 (0.38–1.14)	0.136
Marital status								
Married	1.0 (ref)						1.0 (ref)	
Single	0.66 (0.43–1.03)	0.069	0.88 (0.59–1.33)	0.559	0.91 (0.57–1.45)	0.693	0.91 (0.55–1.51)	0.723
Education								
High school diploma or less	1.0 (ref)		1.0 (ref)		1.0 (ref)		1.0 (ref)	
Some college	1.35 (0.74–2.46)	0.335	0 0.99 (0.58–1.69)	0.975	1.50 (0.81–2.80)	0.194	0.95 (0.48–1.87)	0.878
College graduate or more	3.81 9 (0.85–16.98)	0.080	0 0.57 (0.17–1.88)	0.359	1.38 (0.32–5.90)	0.660	1.44 (0.33–6.34)	0.630
Age								
18–29 years	1.0 (ref)		1.0 (ref)		1.0 (ref)		1.0 (ref)	
30–49 years	0.88 (0.56–1.49)	0.641	0.73 (0.45–1.18)	0.199	0 0.82 (0.49–1.38)	0.452	0.82 (0.47–1.44)	0.492
50 and older	0.90 (0.49–1.61)	0.712	0.54 (0.32–0.93)	0.026	0 0.55 (0.29–1.02)	0.056	0.51 (0.26-1.00)	0.052
Previous cervical cancer screening								
Yes	1.0 (ref)		1.0 (ref)		1.0 (ref)		1.0 (ref)	
No	0.39 (0.22–0.72)	0.002	0.95 (0.55–1.64)	0.858	0 0.66 (0.35–1.25)	0.205	0.38 (0.21–0.72)	0.003

Independent variables	Male physicians		Transportation		Language barriers			
	Adjusted OR (95% CI)	*P* value	Adjusted OR (95% CI)	*P* value	Adjusted OR (95% CI)	*P* value		
Race/ethnicity								
White	1.0 (ref)		1.0 (ref)		1.0 (ref)			
Black	0.76 (0.44–1.35)	0.361	1.30 (0.70–2.43)	0.393	0.34 (0.14–0.84)	0.020		
Hispanic	2.16 (1.32–3.55)	0.002	1.00 (0.56–1.79)	0.393	4.72 (2.62–8.50)	0.000		
Marital Status								
Married	1.0 (ref)				1.0 (ref)			
Single	0.74 (0.49–1.15)	0.188	0 0.71 (0.43–1.18)	0.186	0.59 (0.36–0.96)	0.035		
Education								
High school diploma or less	1.0 (ref)		1.0 (ref)		1.0 (ref)			
Some college	1.44 (0.79–2.62)	0.233	0 0.92 (0.46–1.87)	0.836	0.77 (0.33–1.78)	0.539		
College graduate or more	2.22 (0.56–8.89)	0.259	0 0.27 (0.03–2.31)	0.234	0.97 (0.16–5.81)	0.978		
Age								
18–29 years	1.0 (ref)		1.0 (ref)		1.0 (ref)			
30–49 years	0.75 (0.46–1.25)	0.274	1.09 (0.59-2.00)	0.776	1.22 (0.64–2.34)	0.545		
50 and older	0.63 (0.35–1.13)	0.121	0 0.97 (9 0.49-1.94)	0.950	1.22 (0.59–2.50)	0.580		
Previous cervical cancer screening								
Yes	1.0 (ref)		1.0 (ref)		1.0 (ref)			
No	0.71 (0.39–1.29)	0.258	0 0.42 (0.22–0.79)	0.008	1.11 (0.50–2.47)	0.798		

Identified barriers also varied with age, marital status and previous cervical cancer screening. Women who were older than age 50 had reduced odds of identifying fear of finding cancer (OR 0.54, 95% CI 0.32–0.93) as a barrier compared to women younger than 50. Women who were single were less likely to report English language insufficiency (OR 0.59, 95% CI 0.36–0.96) as a barrier to screening compared to women who were married. Women who had never been screened for cervical cancer had reduced odds of agreeing that feeling embarrassed (OR 0.39, 95% CI 0.22–0.72), lack of transportation (OR 0.42, 95% CI 0.22–0.79), and forgetting to schedule appointments were barriers to screening (OR 0.38, 95% CI 0.21–0.72).

## Discussion

The goal of this study was to assess knowledge of cervical cancer risk factors, and identify perceived barriers to screening among a population of uninsured women eligible for free cervical screening and diagnostic procedures. We examined correlates of cervical cancer risk factor knowledge as well as socio-demographic variations in perceived barriers to screening. In this discussion, we focus on findings from descriptive analyses of responses to questions assessing risk factor knowledge; and the role of race/ethnicity, age, and previous cervical cancer screenings to better understand these barriers.

### Cervical Cancer Risk Factor Knowledge

In the present study, many of the respondents were aware of several key risk factors for cervical cancer. More than 70% were aware of an increased risk from unprotected sex or sexually transmitted infections, but only 61% correctly identified multiple sexual partners as a risk factor. Less than half of our sample were aware that smoking increases risk; but even fewer recognized long-term use of birth control and multiple births as risk factors for cervical cancer. The results show a relatively high level of awareness about the risk factors for cervical cancer that are attributed to sex, but lower knowledge about non-sexual risk factors such as smoking cigarettes, long term use of birth control pills, and multiparity. The higher awareness of risk factors attributable to sex may be due to increasing publicity around the risk of human papilloma virus and unsafe sex. Nonetheless, the lower awareness of non-sexual risk factors shows gaps in the risk factor knowledge that may be addressed through patient education.

Total risk factor knowledge scores varied significantly with educational attainment, but not with race, marital status, age or previous cervical cancer screening. The association between educational attainment and risk factor knowledge has been corroborated by other studies. A study in Chicago found that health literacy was a stronger predictor of knowledge of cervical cancer risk factors than race and ethnicity [20]. Though not consistent with our results, another study found that women who were married with children, and women who previously had abnormal pap smear results had better knowledge of cervical cancer risk factors [27]. The significant positive correlation between total knowledge scores and literacy underscores the importance of health education in raising awareness about these risk factors.

Although many respondents knew that regular Pap smears could result in early detection of cervical cancer, only 52% of the age-appropriate sample had received Pap smears within the past two years. This relationship between knowledge of cervical cancer risk factors and poor screening compliance highlights the presence of other potential barriers that might impede screening, despite adequate knowledge. Among the three racial/ethnic groups, knowledge scores were lowest among Hispanics and highest among Blacks, although in multivariate analyses Blacks were significantly more likely to report lack of knowledge as a barrier to screening.

### Role of Race/Ethnicity on Barriers Identified

The results showed socio-demographic variations in the participants’ identification of perceived barriers. Multivariate analyses showed that Hispanics had higher odds of identifying fear of finding cancer, male physicians, and language as barriers to screening. These are personal and cultural barriers [5] that may reflect Hispanics’ cultural values, cultural assimilation, and perception of physician-patient relationships. Among Hispanics, fear of finding cancer also has been corroborated by other studies [8, 18, 28] and is not limited to cervical cancer but also extends to breast and colorectal cancers [29, 30]. Likewise, studies have suggested that Hispanics and Blacks were more likely to have fatalistic beliefs about cancer, and would rather not know that they have cancer, believing that nothing could be done to prevent it; and these fatalistic beliefs significantly impacted their choice to get screened [7, 31]. In our study, Blacks were also likely to state fear of finding cancer as a barrier; however, this finding was not statistically significant.

Not surprisingly, Hispanics were more likely to identify language as a barrier. This was expected, given that 58% of Hispanics in our sample identified their primary language as Spanish, and 53% of those who identified Spanish as their primary language agreed that language was a barrier to screening. Other studies have confirmed a link between ability to communicate in English as a primary language as a barrier to screening [8, 32]. A study which focused exclusively on Hispanic women found that even women who had identified English as their primary language preferred to communicate in Spanish with their providers [10]. More so, data from the 2000 National Health Interview Survey revealed that Hispanics who were less proficient in English language were less likely to obtain physician recommendation for Pap smears [33], which is a strong predictor of receiving cervical cancer screening, regardless of race.

Studies have identified physician gender as a barrier to screening uptake and compliance [18, 28, 34]. Our results showed that Hispanics were twice as likely to identify male physicians as a barrier to screening. In agreement with our results, Hispanics have been reported to be more likely to report feelings of nervousness and embarrassment in discussing their sexual activity, and being examined by a male physician [28, 35]. Other studies found that older Hispanic women were more likely to report being embarrassed, regardless of the gender of the examining physician [34, 35].

### Role of Age on Barriers Identified

Barriers to cervical cancer screening identified by older women in previous studies include lack of physician recommendation [12], fewer clinic visits [13], chronic diseases [23], prior negative experiences with the healthcare system [36]; and language barriers for older, less acculturated Hispanics [10, 13]. In this study, women older than age 50 were less likely to identify fear of finding cancer as a barrier, compared to younger women.

Regardless of race/ethnicity, cervical cancer screening compliance is reportedly higher among younger women, which makes early-stage detection of cervical cancer more likely [5]. Moreover, other studies have reported that young Black women are more likely to be screened compared to their white peers, but older Black women are less likely to be screened, and are therefore more likely to be diagnosed with late stage cervical cancer [5, 37].

### Role of Previous Screening

In the present study, women who had never been screened were less likely to identify personal barriers such as feelings of embarrassment, and structural barriers such as lack of transportation and forgetting to schedule an appointment, as impediments to screening. This is not consistent with previous findings that suggest that personal barriers prevent initial screening, but structural barriers limit regular screening [18].

Despite these barriers, many minorities and low income women still desire and seek preventive care [24]. Self-identified barriers may potentially be reduced through provision of culturally appropriate prevention education and patient care. In addition to conducting free or subsidized safety-net screenings and diagnostics for medically underserved women, equipping the next generation of primary care physicians with tools for culturally relevant education and patient care is critical to improve screening rates for safety-net populations.

### Limitations

This study has several limitations. First, the survey was provided only to women who were purposefully presenting for screening and may therefore, already have some knowledge about screening for cervical cancer. The barriers identified may differ for women who have never presented for screening. Also, given that the sample was made up of mostly uninsured women with low educational attainment, our results may not be generalizable to an insured and more educated population. The perception of barriers is self-reported, and it is difficult to measure the validity of these responses.

## Conclusion

This study presents the level of awareness of cervical cancer risk factors, especially among the uninsured. Researchers may use this understanding to isolate risk factors that are most misunderstood, and explore the most commonly cited barriers in planning cancer screening programs. These programs would focus on education about risk factors and improving opportunities for women to obtain screening.

Over the years, the numbers of women who have received cervical cancer screening has increased, but it will be difficult to sustain this progress if key barriers are not addressed. An understanding of the barriers and facilitators of cervical cancer screening can enable healthcare providers and the public health workforce to be sensitive to the unique needs of these populations as they work towards overcoming these hurdles. Since women who have unfavorable attitudes toward cervical cancer screening might exhibit the same behavior with other cancer screening types, such as breast mammograms [38], helping women overcome these barriers might make them more accepting of other preventive healthcare measures.
